# 25179 The Community Mentor for Scholars Program: Pilot Program Results

**DOI:** 10.1017/cts.2021.602

**Published:** 2021-03-31

**Authors:** Angela Merrifield, Michelle Lamere, Sheila Riggs, Nicole Bowman, Jayne A. Fulkerson, Janet Shanedling, Sara Rohde, David H Ingbar

**Affiliations:** University of MN

## Abstract

ABSTRACT IMPACT: Strengthening investigator and community engagement to improve human health OBJECTIVES/GOALS: Community Engagement is one of the 14 core competencies for CT research defined by the CTSA Education Core Competency Work Group. To meet this, the UMN CTSI created the Community Mentor for Scholars Program with goals to: 1) train Scholars to engage stakeholders; and 2) provide community with formal mentoring training and linkages to researchers at UMN. METHODS/STUDY POPULATION: The CM Program was implemented over 12 months and includes four components. One, Scholars were trained in stakeholder identification and working with a community mentor (CM) through two seminars presented by expert faculty and staff. Two, CMs were identified, recruited, and matched with Scholars through a collaborative effort of our CTSI Education and Community Engagement cores. Three, Scholars and CMs learned about the program from a 2-hour Kick-Off event. Four, CMs and Scholars each completed four online modules developed through an NCATS administrative supplement. Scholar-CM pairs met at least four times to plan and hold a bi-directional ‘Community Conversation’ with an audience of key stakeholders convened by the CM. The CM Program was evaluated through in-person interviews. RESULTS/ANTICIPATED RESULTS: In 2019-2020, CTSI initiated the pilot program with four KL2 Scholar - CM pairs. Two pairs did not complete the program due to time pressures, a parental leave, and the COVID-19 pandemic. Feedback from the two Scholar - CM pairs was positive, specifically:

CMs reported the training modules were useful, resulting in better understanding of CTSI research programs and increased capacity to mentor

Scholars felt the interactions with CMs positively impacted their future research

Mentors supported experiential learning, offered insight on community perspectives, and successfully facilitated community engagement principles. DISCUSSION/SIGNIFICANCE OF FINDINGS: The second cohort launched in late 2020 with inclusion of TL1 Scholars. They will be matched with CMs in spring 2021. After Cohort 2 completion, the program design and materials will be updated based on evaluation results from scholars and mentors and then will be piloted with select CTSAs before sharing across the CTSA consortium.

